# Severe Bilateral Vertebral Arterial Disease Masquerading as Vertigo

**DOI:** 10.7759/cureus.50410

**Published:** 2023-12-12

**Authors:** Juwayria A Ahmed, Khudheeja A Ahmed, Mohammed Habeeb Ahmed

**Affiliations:** 1 Department of Research, KAAJ Healthcare, San Jose, USA; 2 Department of Cardiology, KAAJ Healthcare, San Jose, USA

**Keywords:** noninvasive imaging modalities, central vertigo, vertebrobasilar insufficiency (vbi), vertebral artery stenosis, bilateral vertebral artery disease

## Abstract

Vertebral artery stenosis (VAS), manifesting as vertebrobasilar insufficiency (VBI), results from the narrowing of the vertebral artery's lumen because of diverse vascular pathological processes, leading to various clinical presentations. We present the case of a 71-year-old male who experienced vertigo for almost two years and was ultimately diagnosed with severe bilateral VAS. Despite initial management for vertigo, the patient's symptoms persisted, prompting a referral to a cardiologist. Several assessments were performed, including an MRI of the brain, which ruled out acute intracerebral hemorrhage or infarction. After some visits to the cardiologist’s office, the patient was referred to a neurologist who conducted a magnetic resonance angiography (MRA) of the neck, which showed an occlusion of the left vertebral artery at the origin and a patent right vertebral artery. Because of worsening symptoms of vertigo and the results of the MRA, the patient underwent invasive angiography that confirmed the occlusion of the left vertebral artery and also revealed severe stenosis of the right vertebral artery. This case report discusses an unusual presentation of VAS with vertigo as the primary symptom, emphasizing the importance of recognizing seemingly minor symptoms as manifestations of the underlying vascular pathology that requires careful evaluation. Furthermore, this case emphasizes the limitation of relying solely on noninvasive imaging for diagnosis as, in this instance, noninvasive imaging failed to detect the severe stenosis of the right vertebral artery, which was revealed by invasive angiography. Finally, this case report underscores the significance of collaboration across several disciplines, such as cardiology, neurology, and radiology, as well as endovascular medicine in diagnosing and managing atypical manifestations of complex conditions.

## Introduction

Vertigo is a common clinical complaint encountered by healthcare providers and is characterized by the illusion of rotation caused by asymmetry in neural activity between the left and right vestibular nuclei [1]. The causes of vertigo are broadly categorized into either peripheral or central, with over 90% of cases being attributed to peripheral causes [2]. This classification indicates dysfunction in the peripheral vestibular or central vestibulocerebellar system, respectively. The vertebrobasilar arterial system supplies blood to the brainstem, cerebellum, and peripheral labyrinths, and they may become occluded because of factors such as atherosclerosis or embolism, leading to either central or peripheral vertigo, depending on the specific artery affected [2]. Common peripheral causes of vertigo include benign positional vertigo (BPPV), also known as benign paroxysmal positional vertigo, as well as inflammation of the vestibular nerve (neuronitis) [3]. Additionally, head injury, swelling of the inner ear (labyrinthitis), and certain medications such as aminoglycoside antibiotics, cisplatin, diuretics, or salicylates may harm the inner ear structures and cause vertigo [3]. Central causes, while more uncommon, can be more serious. Central vertigo often originates from ischemia in the cerebellum, brainstem, or vestibular nuclei, especially in the elderly with risk factors for vascular disease [2]. Acute demyelination such as in multiple sclerosis, medication toxicity (e.g., anticonvulsants), infection, trauma, brain tumors, migraine, and blood vessel disease are other causes of central vertigo [2,3]. 

Effectively navigating the diagnostic landscape of vertigo requires distinguishing between peripheral and central causes, which can be difficult. Peripheral causes predominantly present as vestibulocochlear problems such as vertigo, tinnitus, and/or hearing impairment, with exacerbation of symptoms by sudden movements and positional changes [4]. In contrast, features suggesting a central cause include the absence of inner ear symptoms, bilateral or vertical nystagmus not suppressed by visual fixation, and the presence of concomitant brainstem or cerebellar signs [1]. Notably, isolated episodes of vertigo lasting over three weeks are rarely attributed to vertebrobasilar disease [4]. This case report outlines the presentation of severe bilateral vertebral arterial stenosis in the ostial location in a 71-year-old patient experiencing vertigo for almost two years.

Vertebral artery stenosis (VAS) is the constriction of the vertebral artery's lumen. The clinical presentation of VAS depends on the location of the vertebral artery that is affected [5,6]. The vertebral artery, usually the first major branch of the subclavian artery on both sides of the body, can be anatomically divided into four segments (V1-V4) [5]. The extracranial vertebral artery consists of segments V1-V3, while segment V4 is intracranial. VAS most commonly occurs in the V1 segment, referred to as the proximal or ostial segment, which spans from its origin branching off the subclavian artery to the transverse foramen of the sixth cervical vertebrae [5]. V2 spans from the sixth to the second cervical vertebra, and V3 extends from the second cervical vertebra to the foramen magnum. The intracranial segment, V4, spans from the foramen magnum to the formation of the basilar artery. Additionally, the vertebral artery supplies blood to the circle of Willis, which is a vital circulatory anastomosis ensuring collateral circulation between the anterior and posterior cerebral circulation, maintaining continuous blood flow to the brain [5]. VAS arises from factors such as arterial calcification, atherosclerotic lesions, dissections, fibromuscular dysplasia, giant cell arteritis, neurofibromatosis, and bony compressions [5]. Symptoms include vertigo, tinnitus, diplopia, headache, hypokinesis, hearing disorders, dizziness, vomiting, loss of vision, ataxia, numbness, and weakness affecting both sides of the body [4,6]. If unmanaged, VAS may lead to severe outcomes such as cerebrovascular infarctions or strokes, vertebrobasilar insufficiency, and sudden death [5].

The variability and common occurrence of tortuosity in the vertebral artery, especially in the V1 segment, poses imaging and diagnostic challenges. Intra-arterial angiography (IAA) is considered the current gold standard for diagnosing VAS because of its high diagnostic accuracy [5]. However, IAA is also considered the riskiest, most invasive, expensive, and time-consuming diagnostic technique for VAS, leading to increased utilization of noninvasive alternatives such as duplex ultrasonography (DUS), computed tomography angiography (CTA), and MRA [5].

DUS is the standard noninvasive initial screening for VAS, and it is considered safe, accurate, and cost-effective [5]. However, DUS faces limitations in accurately depicting the degree of vascular stenosis, especially at the origin of the vertebral artery, because of its tortuosity, small diameter, and perpendicular position, resulting in successful imaging in only around 60% of subjects [5]. DUS effectiveness has been improved to over 80% by incorporating color flow imaging and Doppler parameters [5,7]. Transcranial Doppler ultrasound (TCD) detects intracranial VAS with a sensitivity of up to 80% and a specificity ranging from 80% to 97% when compared to IAA [7]. However, it tends to underestimate the degree of stenosis in over half of the cases and may miss occlusion of vessels [7].

Helical or spiral CTA is a noninvasive means of imaging the extracranial vertebral artery without the risks associated with catheter angiography as in IAA. One study involving 24 patients with vertebrobasilar ischemic symptoms found that CTA successfully visualized the vertebral artery origin and detected the extracranial vertebral artery stenotic lesions identified by IAA in all cases [7]. Moreover, it demonstrated potential diagnostic advantages in distinguishing between "kinked" and genuinely atherosclerotic stenotic vessels [7]. However, CTA tends to underestimate the prevalence and extent of stenosis in the ostial vertebral artery and also faces challenges in accurately determining the lumen diameter of extensively calcified arteries [5].

MRA is a noninvasive imaging technique used for assessing VAS that visualizes hemodynamic flow by discriminating between flowing spins in blood and those in stationary tissue [8]. There are two primary classes of MRA methods: time-of-flight (TOF) methods and phase contrast methods [8]. TOF methods enhance the magnetic resonance imaging signal by relying on the amplitude of moving spins in the blood, while phase contrast methods derive the signal from the phase accumulated by the flowing spins [8]. The pooled data of a retrospective study conducted by Wentz et al. examining 60 basilar and 106 intracranial vertebral arteries and a prospective study by Strotzer et al. involving 40 patients shows that TOF MRA has a sensitivity of 71.4 (95% CI (confidence interval) 47.8 to 88.7) and a specificity of 95.1 (95% CI 91.1 to 97.6) for 50-99% stenosis and a sensitivity of 100 (95% CI 75.3 to 100) and a specificity of 100 (95% CI 97.5 to 100) for occluded arteries [9-11]. However, TOF MRA faces multiple challenges in assessing VAS, especially in imaging ostial VAS because of factors like breathing movements and large vessel pulsations, potentially leading to an overestimation of ostial VAS [9,12]. Furthermore, a review conducted at the New England Medical Center involving 62 patients found that TOF MRA might fail to detect lesions at the intricate junction between the intracranial and extracranial segments of the vertebral artery [13]. Additionally, its reliance on flow-related enhancement makes TOF MRA susceptible to misinterpreting low flow as the absence of a vessel, resulting in a tendency to overreport occlusion in instances of high-grade stenosis [12]. However, the inclusion of contrast agents in contrast-enhanced MRAs (CE-MRAs) enhances the signal-to-noise ratio to mitigate this concern, making CE-MRA a promising alternative for detecting VAS. A meta-analysis conducted by Khan et al. found CE-MRA more sensitive and specific than DUS and non-contrast MRA in detecting VAS [9].

## Case presentation

A 71-year-old male presented to the cardiologist’s office on November 3, 2021, with a range of complaints, including palpitations occurring intermittently for the past few weeks, leg cramps in both calves, particularly at night, fatigue, dizziness, and loss of balance. He was accompanied by his son, who is a practicing primary care physician himself. Orthostatic hypotension was ruled out. He was also seen by the ENT doctor and treated for vertigo by his primary care physician earlier.

He was taking the following medications at the time of the presentation: amlodipine besylate 5 mg daily, aspirin 81 mg daily, atorvastatin calcium 20 mg daily, lisinopril 40 mg daily, and prednisone 10 mg daily. Prior evaluations done earlier in the year included an echocardiogram done on 7/24/21, which showed normal left ventricular size and function, mild to moderate LVH, and minimal valvular abnormalities, including trace AI, trace MR, mild TR, and trace PI. A 24-hour Holter monitor done on 7/13/21 had shown sinus rhythm with heart rates ranging from 61 beats per minute (bpm) to 130 bpm with an average rate of 77 bpm and no arrhythmias.

Further assessments were conducted, including ambulatory cardiac monitoring from 11/19/21-12/03/21, which revealed sinus rhythm with heart rates ranging from 60 bpm to 141 bpm, an average of 84 bpm, occasional premature ventricular contractions (PVCs), and runs of supraventricular tachycardia (SVT). An MRI of the brain with and without contrast done on 11/30/21 ruled out acute intracerebral hemorrhage or infarction. However, symptoms persisted during a follow-up visit on December 8, 2021, with the patient reiterating complaints of palpitations, dizziness, loss of balance, leg cramps, and fatigue.

A noteworthy update occurred during a follow-up visit on 04/06/22, after the patient's return from Mexico, where the patient reported that his symptoms, including ataxia, had resolved. By July 6, 2022, he was feeling better overall but continued to experience occasional dizziness. Subsequently, during a visit on April 12, 2023, the patient reported new symptoms, including episodes of chest pain, which were intermittent, substernal in location, radiating to the left arm, and associated with tingling and numbness in the left arm, particularly at night. An echocardiogram on 04/18/23 showed a left ventricle with normal size and function, an ejection fraction (EF) of 60%, LVH, E/A reversal, and a left atrium at the upper normal limit. Minimal valvular abnormalities remained, with trace MR, mild TR, and a right ventricular systolic pressure (RVSP) of 35 mmHg. A 24-hour Holter monitor on the same date demonstrated sinus rhythm with a heart rate ranging from 66 to 120 bpm, with no observed arrhythmias.

The patient returned to the cardiologist’s office on 05/17/23 after having seen a neurologist who performed an MRA without contrast of the neck in the interim. The patient had been experiencing a progressively worsening pattern of symptoms, including episodes of dizziness, loss of balance, marked fatigue, and a notable lack of energy. The MRA of the neck, done on 08/02/23, revealed the following results. Both the right and left carotid arteries, encompassing the common and internal carotid arteries, exhibited patent and unobstructed flow with no evidence of any hemodynamically significant stenosis by NASCET criteria. The right vertebral artery exhibited patent status without evidence of flow-limiting stenosis, dissection, or pseudoaneurysm. The left vertebral artery was occluded at the origin with reconstitution of flow at approximately the level of the carotid bifurcation.

**Figure 1:**
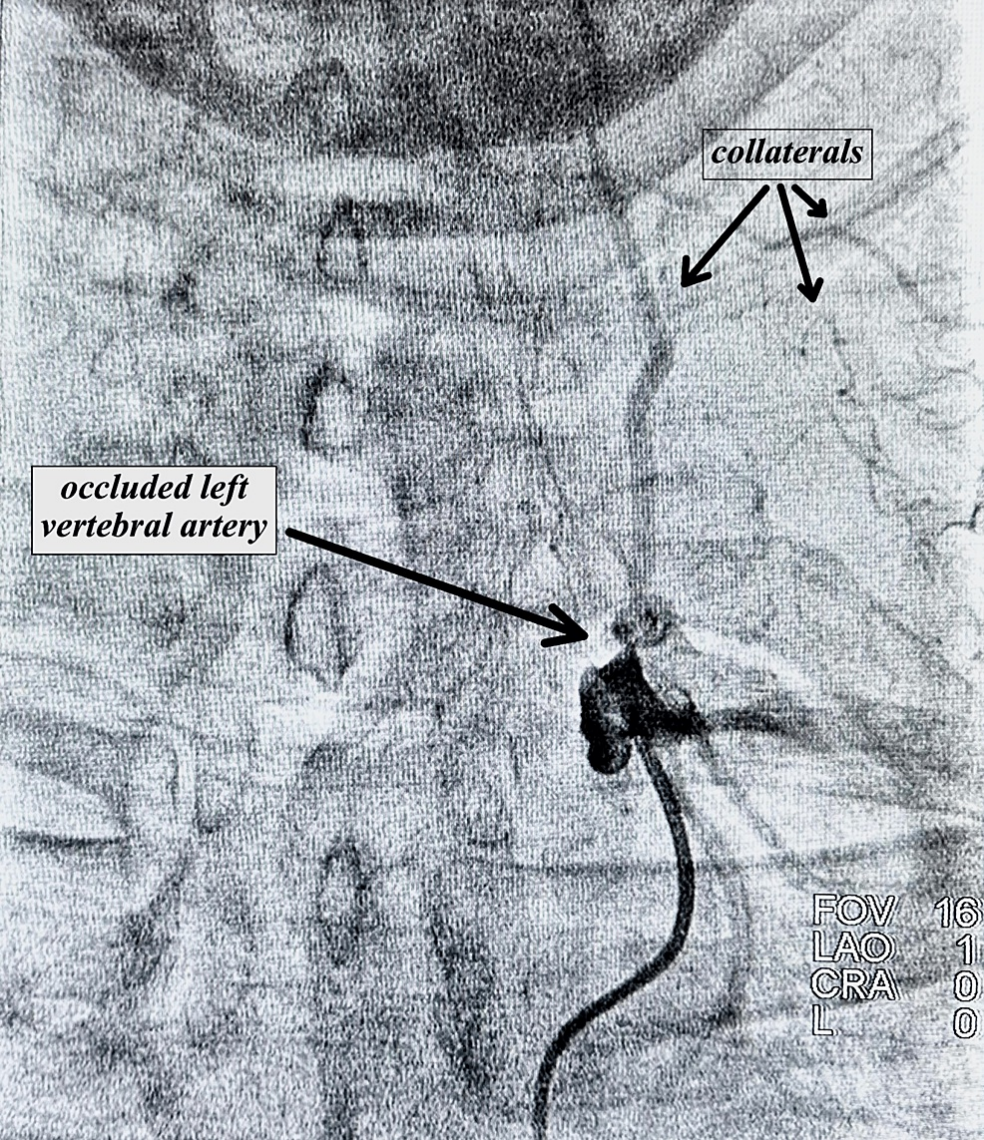
Angiography showing occluded left vertebral artery as well as collaterals

**Figure 2:**
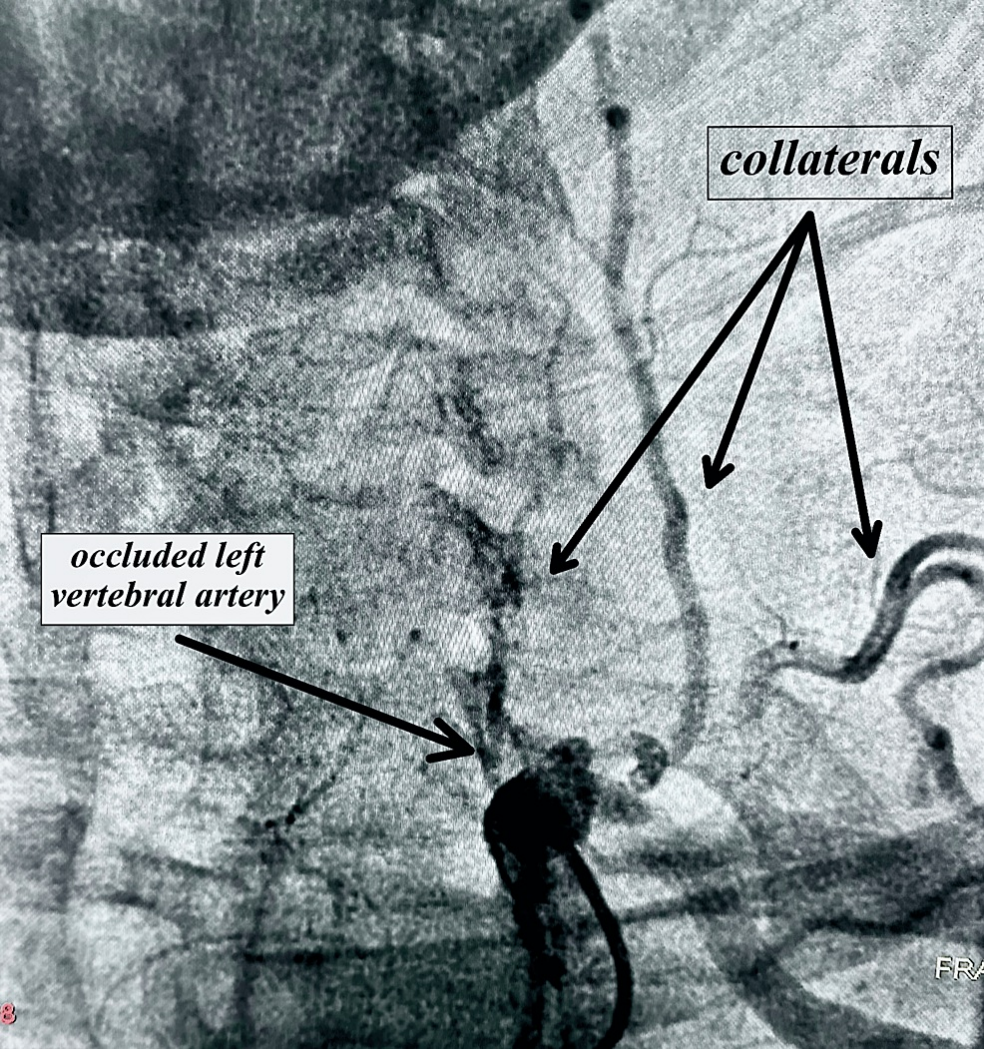
Angiography showing occluded left vertebral artery as well as the extensive collateralization

**Figure 3:**
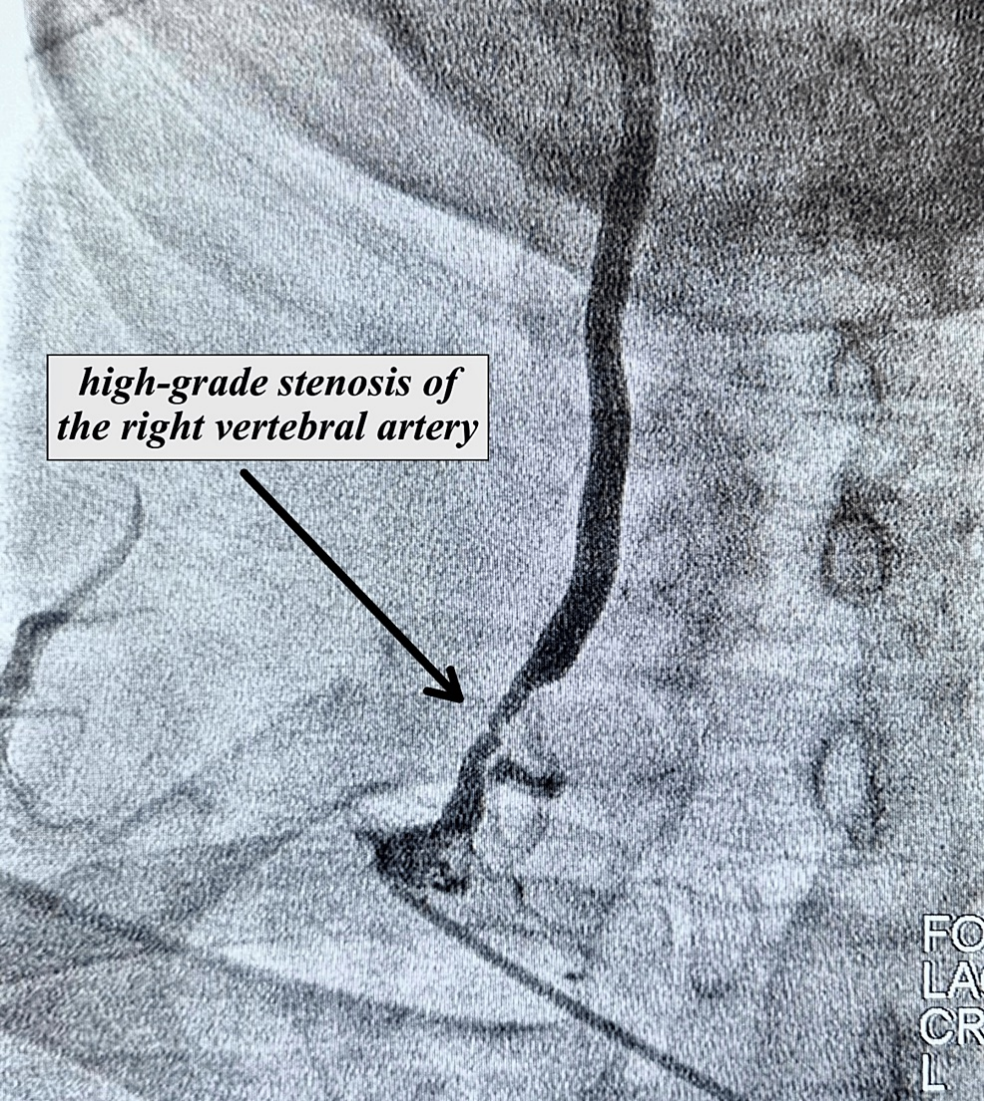
Angiography showing right vertebral artery with 80-90% stenosis

During the 08/16/23 follow-up, the patient reported worsening episodes of dizziness, loss of balance, and fatigue. As a result, a comprehensive evaluation encompassing left heart catheterization and cerebrovascular angiography was performed on 10/03/23 to assess angina, coronary artery disease, and carotid/vertebral circulation. The angiographic results revealed moderate disease of the left anterior descending (LAD) artery, occlusion of the left vertebral artery at its ostium, severe stenosis of 80-90% in the right vertebral artery at the proximal segment, and an EF of 55-60%. Images from the angiography are shown in Figures 1-3 below. In the post-angiogram checkup on 10/11/23, the patient had no procedure-related complaints and felt relatively stable, although he was still experiencing occasional dizziness. The recommended next step was an MRI of the brain with and without contrast, focused on assessing the possibility of any previous strokes. The MRI with and without contrast done on 11/09/23 showed generalized cerebral atrophy with normal cerebral hemispheres, basal ganglia, thalamic nuclei, midbrain, pons, and cerebellum. There was a normal diffusion signal with no bleeding, acute CVA, mass lesion, or midline shift. No abnormal enhancement was noted on post-contrast imaging. There was paranasal sinusitis.

## Discussion

VAS refers to the narrowing of the vertebral artery's lumen, a condition that can occur unilaterally or bilaterally, with severity ranging from moderate to severe stenosis or complete occlusion [5]. Diagnosing VAS proves challenging for two main reasons. First, the symptoms of VAS are varied, including vertigo, diplopia, dizziness, loss of consciousness, dysphagia, dysarthria, nausea, ataxia, nystagmus, and numbness, many of which are frequent clinical complaints that more commonly have less serious causes, making it difficult to recognize these symptoms as indicative of VAS [5]. For example, vertigo has an estimated prevalence between 20% and 56% within the general population, with over 90% of the cases due to peripheral causes [2,14]. The tendency for VAS to go unnoticed is exemplified in our report, which describes a case of a patient who presented with vertigo and was diagnosed with severe bilateral VAS almost two years after the initial complaint. Interestingly, the patient’s son who lives with him is a practicing primary care physician, demonstrating the difficulty of recognizing symptoms of VAS. Moreover, VAS may be asymptomatic as well. A study involving 3,717 patients revealed that 7.6% of individuals with symptoms of atherosclerotic arterial disease exhibited asymptomatic VAS of greater than 50% or occlusion [15]. Over an average follow-up period of 4.6 years, five instances of posterior circulation ischemic stroke were observed among the 282 patients diagnosed with asymptomatic vertebral artery origin stenosis [15].

Another noteworthy aspect of this case is the relative stability exhibited by the patient despite total occlusion of the left vertebral artery and high-grade stenosis of the right side. Generally, symptomatic VAS left undiagnosed has the potential to lead to severe outcomes such as cerebrovascular accidents (CVA), vertebrobasilar insufficiency (VBI), and sudden death [5]. The endovascular specialist hypothesized that the formation of collaterals and normal carotid and intracerebral circulation are likely responsible for the patient's relative stability. 

The intricate, tortuous, and variable anatomy of the vertebral artery makes it difficult to obtain accurate imaging, presenting a second challenge in the diagnosis of VAS [5]. Intra-arterial angiography (IAA) is the most accurate imaging technique and is therefore considered the gold standard. However, because of concerns about its invasive nature and associated risks, healthcare professionals are increasingly turning to noninvasive alternatives such as DUS, CTA, MRA, and CE-MRA [5]. A systematic literature review conducted by Khan et al. comparing the precision of DUS, MRA, and CTA in identifying severe VAS with IAA as the reference standard found that CE-MRA had the highest overall sensitivity followed by CTA, then color DUS, and then finally DUS without color [9]. The review evaluated the sensitivity and specificity of each noninvasive method for varying stenosis categories (50-99%, 50-69/70%, 70-99%, and occlusion), with the diagnosis of occlusion consistently demonstrating the highest sensitivity and specificity across all categories [9]. Particularly, for occluded arteries, CE‐MRA had a sensitivity of 89.5% and specificity of 99.6%, MRA had a sensitivity of 100% and specificity of 100%, color duplex had a sensitivity of 83.3% and specificity of 100%, and duplex without color had a sensitivity of 98.8% and specificity of 90.8% [9]. For arteries with 70-99% stenosis, CE‐MRA exhibited a sensitivity of 83.3% and specificity of 98.5%, CTA showed a sensitivity of 100% and specificity of 100%, and color duplex demonstrated a sensitivity of 65.2% and specificity of 99.3% [9]. Because of the relatively high sensitivities, CE-MRA and CTA have been suggested as potential alternatives to the gold standard IAA [9]. 

However, this case highlights that relying exclusively on noninvasive procedures, such as MRA, can sometimes lead to incomplete assessments. Remarkably, in this case, the MRA was able to identify only the occlusion in the left vertebral artery while erroneously suggesting that the right vertebral artery was entirely free of disease, despite the substantial 80-90% blockage. This underscores the necessity of turning to invasive angiography to uncover conditions that may elude noninvasive methods. Furthermore, it is noteworthy that while the standard neurological evaluation, as well as the subsequent MRI and MRA procedures, played crucial roles in the diagnostic process, they were individually insufficient in determining the etiology of the patient's condition. In contrast, it was the endovascular assessment that ultimately determined the definitive diagnosis. This emphasizes the importance of collaborative efforts and collective expertise in effectively managing complex cases through comprehensive interdisciplinary assessment. In light of these findings, we strongly recommend that patients facing similar diagnostic challenges consider consultation with an endovascular specialist as an integral component of their clinical management.

## Conclusions

This case exemplifies the clinical importance of recognizing that seemingly innocuous symptoms may be indicative of underlying vascular pathology, necessitating vigilant evaluation and collaboration across multiple specialties. The report highlights the intricacies and difficulties of imaging the vertebral artery and diagnosing VAS, including a comparative analysis of the sensitivity of noninvasive methods against invasive angiography. Through this report, we aim to contribute to the expanding knowledge base of otolaryngologists and neurologists and cardiovascular and endovascular specialists in diagnosing and managing atypical manifestations of VAS.
